# Chromatin features of plant telomeric sequences at terminal vs. internal positions

**DOI:** 10.3389/fpls.2014.00593

**Published:** 2014-11-04

**Authors:** Eva Majerová, Terezie Mandáková, Giang T. H. Vu, Jiří Fajkus, Martin A. Lysak, Miloslava Fojtová

**Affiliations:** ^1^Mendel Centre for Plant Genomics and Proteomics, Central European Institute of Technology and Faculty of Science, Masaryk UniversityBrno, Czech Republic; ^2^Max Planck Institute for Plant Breeding ResearchCologne, Germany; ^3^Institute of Biophysics, Academy of Sciences of the Czech Republic v.v.i.Brno, Czech Republic

**Keywords:** telomere, chromatin, epigenetics, *Nicotiana tabacum*, *Ballantinia antipoda*, histone modifications, DNA methylation

## Abstract

Epigenetic mechanisms are involved in regulation of crucial cellular processes in eukaryotic organisms. Data on the epigenetic features of plant telomeres and their epigenetic regulation were published mostly for *Arabidopsis thaliana*, in which the presence of interstitial telomeric repeats (ITRs) may interfere with genuine telomeres in most analyses. Here, we studied the epigenetic landscape and transcription of telomeres and ITRs in *Nicotiana tabacum* with long telomeres and no detectable ITRs, and in *Ballantinia antipoda* with large blocks of pericentromeric ITRs and relatively short telomeres. Chromatin of genuine telomeres displayed heterochromatic as well as euchromatic marks, while ITRs were just heterochromatic. Methylated cytosines were present at telomeres and ITRs, but showed a bias with more methylation toward distal telomere positions and different blocks of *B. antipoda* ITRs methylated to different levels. Telomeric transcripts TERRA (G-rich) and ARRET (C-rich) were identified in both plants and their levels varied among tissues with a maximum in blossoms. Plants with substantially different proportions of internally and terminally located telomeric repeats are instrumental in clarifying the chromatin status of telomeric repeats at distinct chromosome locations.

## Introduction

Epigenetic mechanisms are involved in the regulation of crucial cellular processes such as gene expression, replication timing, and cell cycle control. Epigenetic regulation is mediated by changes in chromatin structure induced by chromatin remodeling complexes, DNA methylation, posttranslational modifications of histones, or *via* non-coding RNA molecules. As shown previously, telomeres, complex nucleoprotein structures located at the ends of linear eukaryotic chromosomes, are subjects of epigenetic control and their maintenance is influenced by changes in telomeric and subtelomeric chromatin (reviewed in Galati et al., 2013; Fojtova and Fajkus, 2014). In human cells, proper telomere function is crucial for healthy development. Pathological shortening of telomeres leads to genomic instability and serious malfunctions or premature death, such as in dyskeratosis congenita, aplastic anemia, or immunodeficiency centromere instability and facial anomalies (ICF) syndrome. Reactivation of telomere maintenance in terminally differentiated cells is one of the basic premises for cellular immortalization and cancerogenesis.

In epigenetic analyses of telomeric chromatin, it is important to distinguish between terminal and interstitial telomeric repeats (ITRs), i.e., telomeric repeats without a chromosome capping function, since their chromatin states may differ. In *A. thaliana*, 8 regions of ITRs were described on three chromosomes, ranging from 300 bp to 1.2 kb (Uchida et al., 2002). Large blocks of telomeric repeats were recently found in pericentromeric regions of some chromosomes in representatives of the Solanaceae family (He et al., 2012). Interestingly, the most recent findings revealed large blocks of imperfect telomeric repeats in the proximity of centromeres of all *Ballantinia antipoda* (Brassicaceae) chromosomes (Mandaková et al., 2010). In *Nicotiana tabacum*, no detectable ITR regions were observed (Majerova et al., 2011a) while telomere lengths ranged from 20 to 160 kb (Fajkus et al., 1995a; Kovarik et al., 1996).

Methods used for analyses of telomeric chromatin usually cannot distinguish between ITRs and telomeres. Cytosines in CCCTAAA telomeric repeats have been reported to be partially methylated in *A. thaliana*, suggesting their heterochromatic nature (Cokus et al., 2008). In contrast, telomeric chromatin of *A. thaliana* seems relatively “open,” displaying euchromatic histone modifications such as H3K4me3 (Vrbsky et al., 2010). Nevertheless, the epigenetic state of telomeric chromatin in *A. thaliana* is far from being elucidated because, in contrast to results demonstrating the existence of intermediate heterochromatin at telomeres (Vrbsky et al., 2010), the heterochromatic character of ITRs (including cytosine methylation) and the euchromatic nature of genuine telomeres were also reported (Vaquero-Sedas et al., 2011). Our recent findings show that in tobacco cell cultures, disruption of DNA and histone methylation *via* drug treatment does not influence telomere length (Majerova et al., 2011b), while hypomethylation of the *A. thaliana* genome either by genetic manipulation or by drugs leads to significantly shortened telomeres (Ogrocka et al., 2014). Methylation of telomeric DNA thus apparently represents an additional layer of epigenetic regulation of plant telomere maintenance that is absent in yeast and animal models (reviewed in Fojtova and Fajkus, 2014).

To address open questions regarding the nature of plant telomeric chromatin, we have expanded the research to plant models that differ markedly in their telomere/ITR ratios. Here, we compare the epigenetic state and transcription of telomeric repeats of *N. tabacum* with no detectable ITRs and extra-long telomeres, and of *B. antipoda* with a major fraction of telomeric repeats present as ITRs, forming large centromeric blocks, and short terminal blocks of telomeres. These plants with substantially different fractions of internally and terminally located telomeric repeats facilitate studies on the epigenetic status of plant chromatin with telomeric repeats at terminal or interstitial position.

## Materials and methods

### Plant growth

*N. tabacum* and *N. tomentosiformis* seeds were surface sterilized with 90% bleach SAVO (Biochemie group, a.s., active substance: NaClO), and germinated on plates with Murashige–Skoog (MS) medium (Duchefa Biochemicals) supplemented with 1% sucrose and 0.8% plant agar. After 10 days, seedlings were potted into soil and grown under short day conditions (light, 150 mmolm^−2^s^−1^ for 8 h at 21°C; darkness for 16 h at 19°C) for 1 month. Plants were then planted into a greenhouse until they produced seeds. Plants of *B. antipoda* were grown from seeds that originated from Mount Alexander Regional Park, Victoria, Australia (herbarium sheet no. MEL 2280901). *B. antipoda* seeds were surface sterilized with 0.5% NaOCl and germinated on plates with MS medium in 0.8% plant agar. Then seedlings were put into soil and cultivated in a growth chamber under long day conditions (16 h light, 8 h dark).

### Fluorescence *in situ* hybridization (FISH) of a telomeric probe

Actively growing, young roots of *N. tabacum* were pretreated with ice-cold water for 12 h and fixed in ethanol:acetic acid (3:1) at 4°C for 24 h. Entire inflorescences of *B. antipoda* were fixed in ethanol:acetic acid (3:1) at 4°C overnight and stored in 70% ethanol at −20°C until use. Preparation of mitotic and stretched pachytene chromosomes from *N. tabacum* root tips and *B. antipoda* anthers was as described (Mandaková et al., 2010). A telomeric FISH probe was prepared (Ijdo et al., 1991), labeled with biotin-dUTP by nick translation and ethanol precipitated (Mandaková et al., 2010). Probes and chromosomes were denatured together on a hot plate at 80°C for 2 min and incubated in a moist chamber at 37°C overnight. The hybridized probe was detected by avidin–Texas Red (Vector Laboratories) and amplified by goat anti-avidin–biotin (Vector Laboratories) and avidin–Texas Red (Mandaková et al., 2010, 2013). Chromosomes were counterstained with 4′,6-diamidino-2-phenylindole (2 μg/ml) in Vectashield (Vector Laboratories). Fluorescence signals were analyzed and photographed using an Olympus BX-61 epifluorescence microscope and a CoolCube camera (MetaSystems), and pseudocolored/inverted/merged using Adobe Photoshop CS2 software (Adobe Systems).

### Analysis of terminal position of telomeric repeats and telomere lengths

The length of telomeres was analyzed by the terminal restriction fragment (TRF) method, which is based on digestion of genomic DNA by frequently cutting restriction enzyme(s) having no recognition site within the telomeric repeats. Plant tissues were embedded in agarose blocks and high molecular weight DNA was purified as described (Fojtova et al., 2002). Samples were digested by Bal31 nuclease (NEB) to degrade terminal telomeric sequences and then by a mix of restriction enzymes *Nla*IV, *Hae*III, *Hinf*I (all purchased from NEB) to delimit telomeric regions (Fajkus et al., 1998). High molecular weight DNA in agarose blocks was analyzed by pulse field gel electrophoresis and subsequent Southern hybridization with a radioactively labeled pltel-C probe, see below. For *B. antipoda* samples, low molecular weight DNA in solution was ethanol-precipitated and analyzed by conventional agarose electrophoresis followed by Southern hybridization.

### Relative methylation of telomeres in nicotiana species

Protoplasts of young leaves of *N. tabacum* and *N. tomentosiformis* were isolated (Yoo et al., 2007). Cells were exposed to a mixture of 0.125 g Cellulase R10 (Serva); 0.025 g Macerozyme R10 (Serva) and 0.025 g Pectolyase Y-23 (Duchefa Biochemicals) in 10 ml digestion buffer (0.4 M mannitol, 20 mM KCl, 20 mM MES pH 5.7, 10 mM CaCl_2_, 0.1% BSA) for 4 h. Fresh protoplasts were embedded into agarose blocks and processed as described (Fojtova et al., 2002). DNA in agarose blocks was digested with Bal31 nuclease (1U per block) for 15, 45, and 90 min in a total volume of 300 μl. One third of the sample was used for the TRF analysis to check the extent of telomere shortening by Bal31 cleavage. DNA from the rest of the agarose block was isolated using a QIAEX II Gel Extraction Kit (Qiagen). Four hundred ng of DNA was treated with sodium bisulfite (EpiTect Bisulfite Kit, Qiagen) to convert non-methylated cytosine into uracil (Clark et al., 1994) and transferred onto a Hybond XL nylon membrane (GE Healthcare) by vacuum dot blotting (Bio-Rad Dot Blot). As positive hybridization control untreated tobacco DNA, and as negative control pUC19 plasmid DNA were loaded onto the membrane. Membranes were hybridized with radioactively labeled oligonucleotide probes as follows: (i) overnight at 55°C in 0.25 M Na-phosphate pH 7.5, 7% SDS (w/v), 0.016 M EDTA for the probe pltel-C (CCCTAAA)_4_ that was used as the loading normalizer; (ii) overnight at 42°C in ULTRAhyb™–Oligo Hybridization Buffer (Ambion) for the probe DEGENER (TTAGRRT)_4_, R = G or A, to detect the methylated or partially methylated fraction of telomeric repeats (for details see Majerova et al., 2011b). The membrane was washed 3 times in 2 × SSC and 0.1% (w/v) SDS. Hybridization signals were visualized on a FLA-7000 phosphoimager (FujiFilm) and the signal intensity was evaluated by MultiGauge Analysis Software (FujiFilm). To express the relative methylation of cytosines located in the proximal telomere region vs. the whole length telomere, the ratio of DEGENER/loading hybridization signals in Bal31-digested samples was normalized to that of intact telomeres ((Bal31^DEGENER^/Bal31^pltelC^)/(intact^DEGENER^/intact^pltelC^)).

### Analysis of cytosine methylation in *B. antipoda* ITRs by bisulfite sequencing

Two repetitive sequences, Ba493 and Ba576, found in intrachromosomal regions of *B. antipoda* and containing perfect and degenerated telomeric repeats, were chosen for analysis of the level of methylated cytosines in ITRs. Identification of these sequences was based on *B. antipoda* preliminary whole genome sequencing data (A. Pecinka laboratory, Max Planck Institute for Plant Breeding Research, Cologne, Germany; unpublished results). DNA was isolated from *B. antipoda* leaves, flower buds and blossoms (Dellaporta et al., 1983), converted by sodium bisulfite (EpiTect Bisulfite Kit, Qiagen) and amplified by PCR using 493 and 576 primers (Table S1) and MyTaq DNA Polymerase (Bioline) in a program consisting of initial denaturation (94°C, 2 min) and 35 cycles of 15 s at 94°C, 15 s at 47°C for 493 primers and at 52°C for 576 primers, and 10 s at 72°C followed by a final extension (72°C/10 min). PCR products 251 and 264 bp for Ba493 and Ba576, respectively, were cloned using a TOPO TA cloning kit (Invitrogen) and sequenced (Macrogene). Methylation of cytosines located in the respective sequence context was analyzed by the CyMATE software (Hetzl et al., 2007). Statistical evaluation of the data was carried out using a One-Way independent ANOVA test. Seven clones for each tissue were analyzed. To take in account natural repeat sequence variability with respect to consensus sequences, native DNA (without bisulfite treatment) was PCR amplified using 493 and 576 primers (Table S1) and 15 clones per primer pair were sequenced (Figure S1; GenBank accession numbers KJ922958—KJ922987). Results of cytosine methylation analyses were compared with the consensus sequence of clones obtained by PCR using the same primer pairs and unmodified DNA (Figure S1).

### Analysis of histone modifications by chromatin immunoprecipitation (ChIP)

Chromatin of *N. tabacum* and *B. antipoda* (0.9 g) was cross-linked with 1% formaldehyde (Sigma) and ChIP was carried out using the EpiQuik™ Plant ChIP Kit (Epigentek) with the antibodies against H3K9me3 (Millipore), H3K9me2 (Abcam), H3K4me3 (Abcam), H4K20me1 (Millipore), and H3K27me3 (Millipore). Two different conditions were used for sonication of plant material in a Diagenode sonicator (Bioruptor)—13 rounds of pulses (15 s at 160 W, 60 s off) for *B. antipoda;* and 13 rounds of pulses (15 s at 160 W, 60 s off) and 7 rounds of pulses (15 s at 200 W, 60 s off) for tobacco. Immunoprecipitated DNA was dot-blotted on a Hybond XL nylon membrane (GE Healthcare) and hybridized with the radioactively labeled probes pltel-C, Ba493 ITR region BAC clone, and Ba576 ITR region PCR product (the same primers as for bisulfite sequencing analysis were used, Table S1). The pltel-C probe was labeled with γ-^32^P ATP by T4 Polynucleotide Kinase (NEB) and hybridized for 24 h at 55°C. Ba493 and Ba576 probes were labeled with α-^32^P dATP by a DecaLabel DNA Labeling Kit (Fermentas) and hybridized for 24 h at 65°C. After washing the membranes (3 × 30 min in 2 × SSC + 0.1% (w/v) SDS at 55°C for the pltel-C probe; 3 × 30 min in 0.2 × SSC + 0.1% (w/v) SDS at 65°C for Ba493 and Ba576 probes), hybridization signals were visualized on FLA-7000 (FujiFilm).

### Analysis of telomeric transcripts TERRA and ARRET

Total RNA was isolated from plant tissues using the RNeasy Plant Mini Kit (Qiagen) according to manufacturer's instructions. Quality and quantity of RNA were checked by electrophoresis on a 1% (w/v) agarose gel and by UV-absorbance (NanoPhotometr IMPLEN). Five μg of RNA were transferred to the Hybond-N membrane (GE Healthcare) and fixed for 15 min by UV-crosslinking and subsequently for 2 h at 80°C. Membranes were hybridized with the end-labeled telomeric probes pltel-C (CCCTAAA)_4_, pltel-G (TTTAGGG)_4_, for 24 h at 50°C. After washing under low stringency conditions (3 × 15 min in 2 × SSC + 0.1% (w/v) SDS at 50°C), hybridization signals were visualized using a FLA-7000 phosphofluoroimager (FujiFilm) and evaluated using MultiGauge (Fuji Film) software. Then, membranes were re-hybridized with Ba493 and Ba576 probes, respectively, as described above. Before re-hybridization, the radioactive telomeric probe was removed by 0.1% SDS (90°C for 5 min) and membranes were exposed overnight to ensure that there is no residual signal.

## Results and discussion

### Distinct localization of telomeric repeats at *N. tabacum* and *B. antipoda* chromosomes

In our previous cytogenetic study we showed for *B. antipoda*, an endemic Australian cruciferous species (Brassicaceae) with six chromosome pairs (2*n* = 12), that *Arabidopsis*-like telomeric repeats hybridized to chromosome termini and to all centromeres (Mandaková et al., 2010).

**Figure 1:**
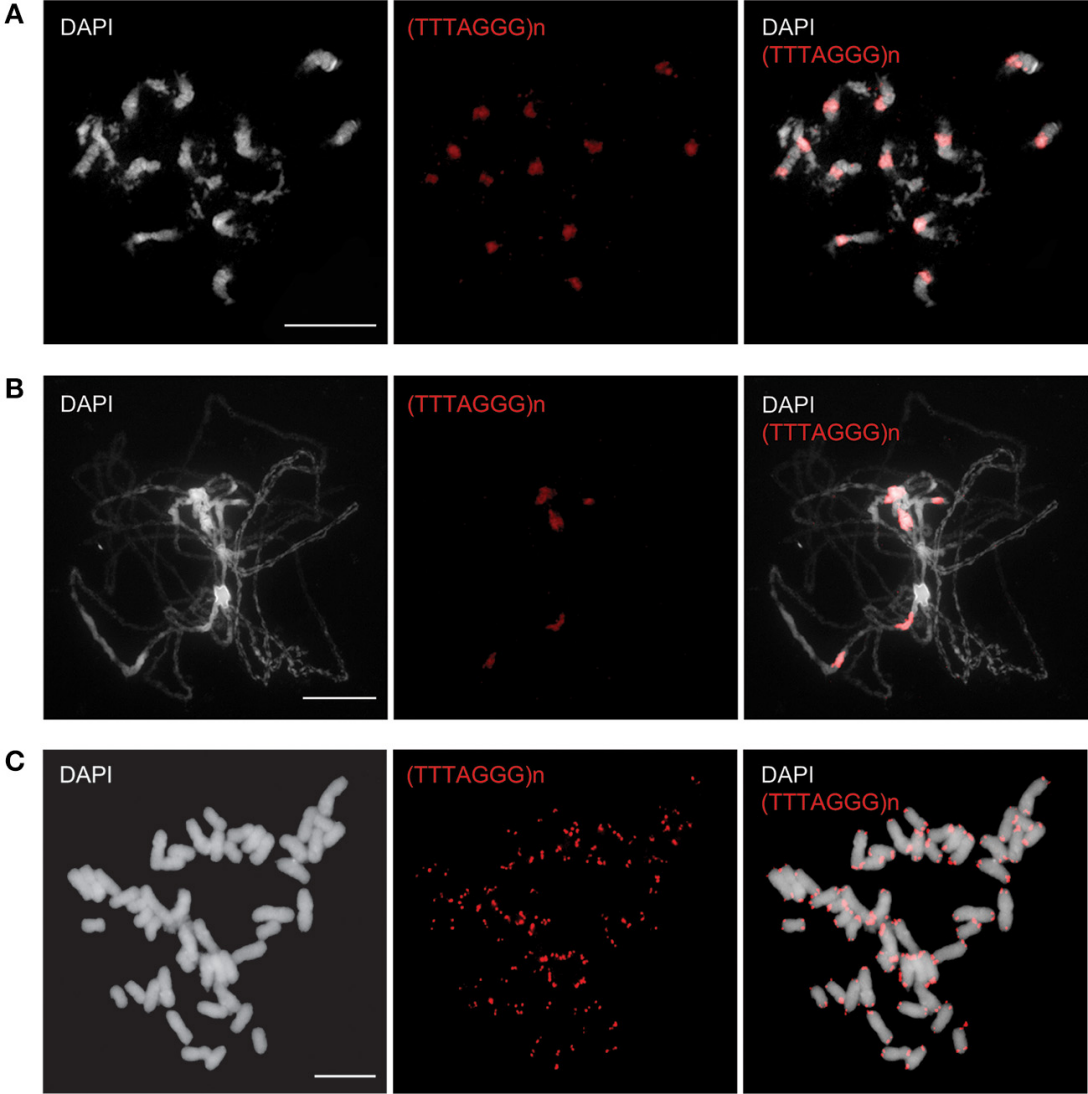
**Fluorescence *in situ* localization of the telomeric repeats in *B. antipoda* and *N. tabacum***. In *B. antipoda*, the telomere repeats (red) hybridize preferentially to centromeres, whereas minor signals at chromosome termini are less prominent on mitotic **(A)** and pachytene **(B)** chromosomes. The exclusive terminal location of the telomere repeats was detected on mitotic chromosomes of *N. tabacum* **(C)**.

**Figure 2:**
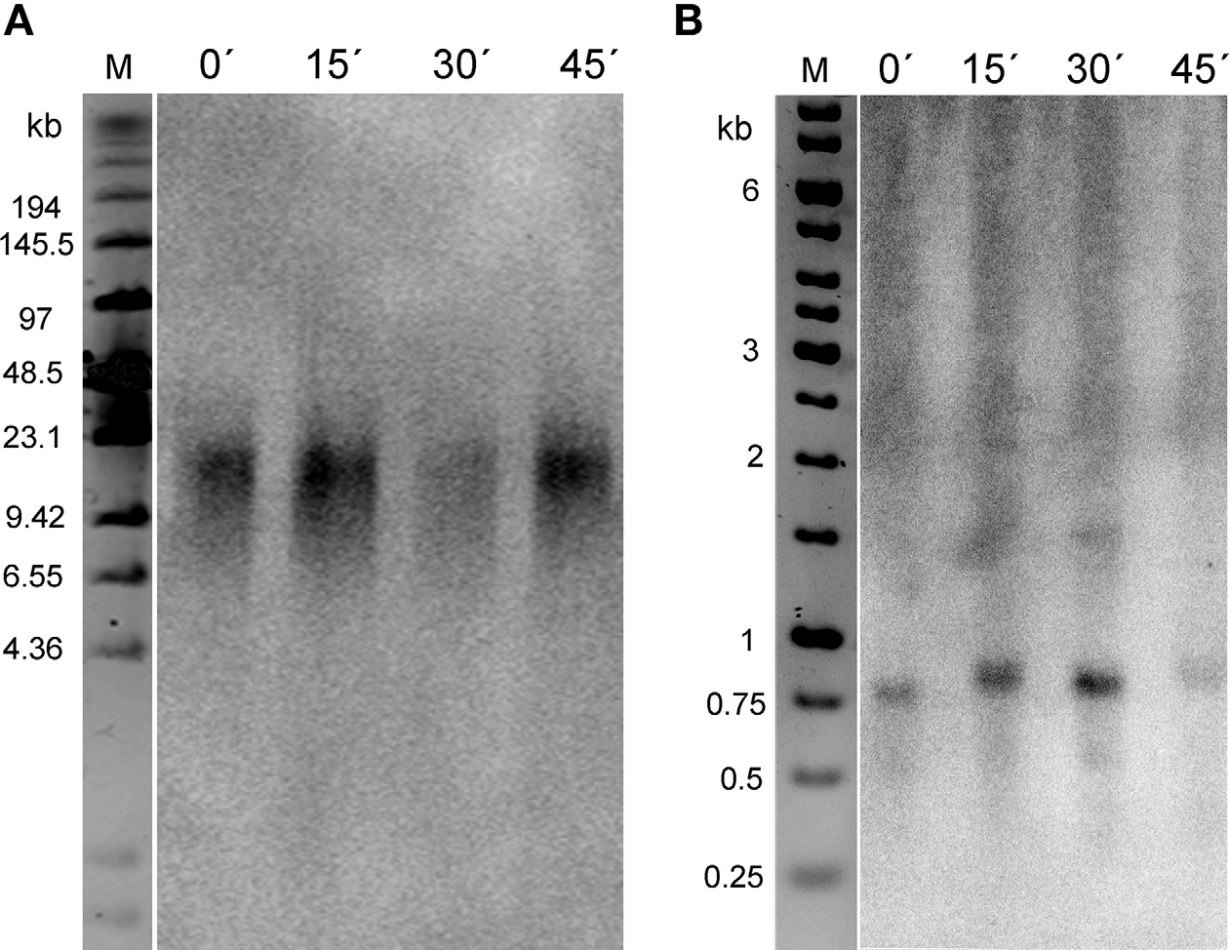
**Internal localization of telomeric repeats in the *B. antipoda* genome. (A)** High molecular weight DNA digested by Bal31 nuclease and a mix of frequently cutting restriction enzymes was analyzed by pulse field gel electrophoresis. Signals after hybridization with the radioactively labeled telomeric oligonucleotide showed resistance of telomeric sequences to Bal31 cleavage. The apparent decrease in signal intensity after 30 min of Bal31 digestion reflects variation in sample loading rather than a change due to the Bal31 treatment (compare to the signal after 45 min of Bal31). **(B)** DNA released into solution in the course of Bal31 digestions was purified and subjected to conventional agarose electrophoresis. The numbers above lines indicate the time of Bal31 digestion in minutes. M, DNA size markers.

To specify the localization of ITRs in *B. antipoda*, we performed FISH on extended meiotic pachytene chromosomes. FISH analysis corroborated the previous report (Mandaková et al., 2010) and showed that ITRs localized to highly condensed centromeric heterochromatin of all chromosomes (Figures 1A,B). Bal31 digestion of high molecular weight DNA confirmed the presence of large Bal31-resistant blocks comprising *Arabidopsis*-like TTTAGGG telomeric repeats in the *B. antipoda* genome (Figure 2A). Approximately 750 bp units of these ITRs were separated by *NlaIV, HaeIII*, and *HinfI* recognition sites (Figure 2B), but a considerable fraction of ITR units was not digested, forming DNA clusters of ca. 20 kb (Figure 2B). Previous cytogenetic and phylogenetic analyses showed that the *B. antipoda* genome has descended from eight ancestral chromosomes (*n* = 8) through an allopolyploid whole-genome duplication event. Due to the extensive reduction of chromosome number (presumably from *n* = 16 to *n* = 6), the six *B. antipoda* chromosomes represent complex mosaics of duplicated ancestral genomic blocks, reshuffled by numerous chromosome rearrangements. Based on knowledge of the exact karyotype structure of *B. antipoda* (Mandaková et al., 2010), we can exclude large ITR tracts as being relics of chromosome rearrangements bringing telomeres into centromere regions.

*N. tabacum* was characterized as a species with long genuine telomeres of relatively heterogeneous length (Fajkus et al., 1995a; Kovarik et al., 1996) and no detectable fraction of internally located telomeric repeats (Figure 1C; Majerova et al., 2011a). In this context, tobacco may be considered as an appropriate model for analysis of epigenetic properties of plant telomeric chromatin, because the content of ITRs in its genome is by orders in magnitude lower (if any) compared to the genuine telomeres and thus allows more straightforward interpretation of results.

### Methylation of cytosines in nicotiana telomeres shows bias with more methylation toward distal telomere position

Methylated cytosines in plant telomeres were detected by whole-genome bisulfite shotgun sequencing (Cokus et al., 2008). Since telomeres are most frequently methylated at the third (inner) cytosine of the CCCTAAA repeat, we designed a degenerate oligonucleotide probe (termed DEGENER) that hybridizes to telomeric sequences methylated at the third cytosine in bisulfite-modified DNA, while the remaining two (outer) cytosines can be either methylated or non-methylated (Majerova et al., 2011b). To evaluate relative telomere methylation in different samples, we normalized the signal obtained with the DEGENER probe to the signal generated by a loading probe (pltel-C) that hybridizes to the G-rich telomeric strand.

**Figure 3:**
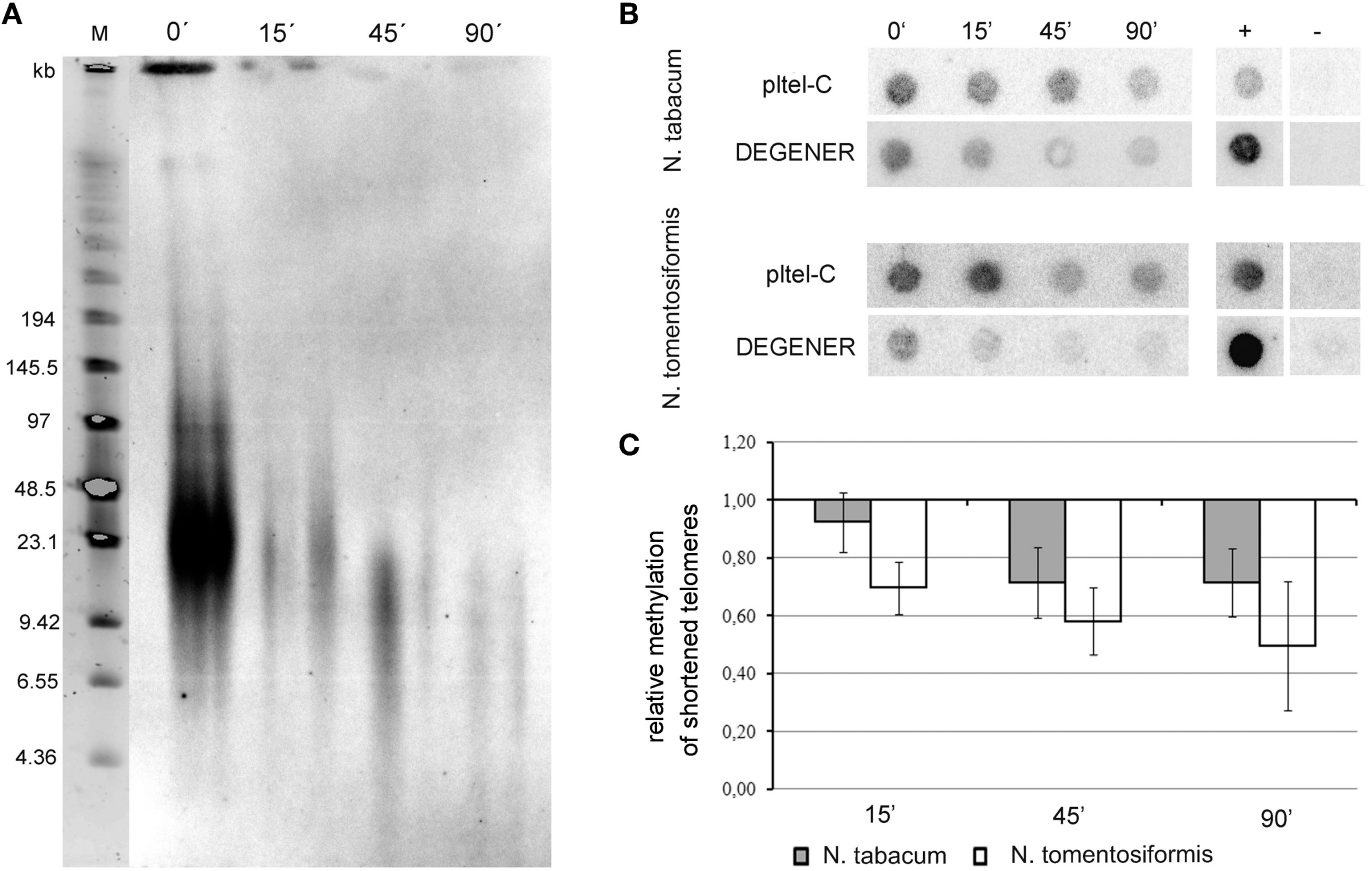
**Relative cytosine methylation in telomeres of *Nicotiana* species. (A)** After Bal31 treatment of *N. tabacum* high molecular weight DNA, TRF analysis was used to control the efficiency of digestion. A loss of the telomere-specific hybridization signal was observed in the course of Bal31 treatment. After 15 min of Bal31 digestion loss of the signal is evident but telomere erosion is not clear, whereas after 45- and 90-min treatments, telomeres were efficiently degraded. Time of Bal31 digestion is given above the lanes. M – DNA size marker. **(B)** Dot-blot analysis of Bal31-digested DNA from *N. tabacum* and *N. tomentosiformis* after treatment with sodium bisulfite. Samples were loaded onto a membrane and hybridized with radioactively labeled probes to detect the total signal of telomeres (loading probe pltel-C complementary to the telomeric G-strand) and the portion of methylated telomeres (DEGENER probe). Time of the Bal31 digestion in minutes is given above the membranes. +, positive hybridization control (tobacco DNA without the bisulfite treatment); −, negative control (DNA from the pUC19 plasmid). **(C)** Relative density of methylated cytosines along telomeres, calculated as the DEGENER/loading hybridization signals ratio. The ratio in Bal31 non-treated samples was arbitrarily taken as 1. Six independent experiments were performed.

Since telomeres in *Nicotiana* species are relatively long (20–160 kb in *N. tabacum* and 20—50 kb in *N. tomentosiformis* Fajkus et al., 1995a; Kovarik et al., 1996) we studied the 5-methylcytosines (5mC) distribution along these telomere repeat tracts. For this purpose, high molecular weight DNA was progressively digested with Bal31 exonuclease. Efficient degradation of telomeres in Bal31-digested samples was checked by (i) TRF analysis that showed loss of the telomere-specific hybridization signal (Figure 3A) and by (ii) hybridization using the loading pltel-C probe. Similarly as in TRF analysis, the telomere-specific signal in Bal31-digested samples was markedly reduced (Figure 3B).

From the ratio of hybridization signals using DEGENER and loading probes (Figures 3B,C), it became evident that in *Nicotiana* species, 5mCs were not uniformly distributed along the telomeres. There was a difference in methylation level at the proximal part of the telomere relative to the whole telomere. In *N. tabacum*, after 90 min of Bal31 digestion, telomeres were degraded to ca. 2/3 of their initial amount, as determined by comparing loading signal intensities in the dot blot analysis (Figure 3B). In this sample, the relative density of methylated cytosines decreased to ~80% (Figure 3C), demonstrating that the level of methylated cytosines is slightly lower in the proximal part of the telomere compared to the average value along the whole telomere.

In *N. tomentosiformis*, the relative decrease in 5mC density toward the proximal part of the telomere was similar as in *N. tabacum* but shifted toward higher relative values corresponding to the shorter total telomere lengths in *N. tomentosiformis*. After 90 min of Bal31 digestion, telomeres were degraded by ~40% (Figure 3B) and the relative density of 5mC in remaining parts of telomeres was ~50% compared to the sample with intact telomeres (Figure 3C). The level of 5mC in *N. tomentosiformis* is thus markedly higher at the distal part of telomeres.

We conclude that both *Nicotiana* species show remarkable similarities in increased relative methylation density at distal telomere regions. Strikingly, in tobacco chromosomes, the distal telomere domain, which is maintained by the combined action of telomerase and the conventional replication machinery, showed a higher 5mC level than the proximal telomere domain which is (i) adjacent to the densely methylated subtelomeric heterochromatin (Fajkus et al., 1995b), is (ii) replicated in a conventional way and is (iii) closer to the presumed transcription start site of TERRA transcripts (see below). According to recent findings, transcribed DNA regions with asymmetry in C/G distribution between the strands (e.g., telomeres) can be protected from methylation by formation of RNA-DNA hybrids (R-loops) when the C-rich DNA strand is transcribed and G-rich RNA is produced (Ginno et al., 2012). This situation could happen in the case of telomeres producing TERRA transcripts (G-rich). These transcripts elongate toward distal parts of telomeres and may not span the entire telomere length.

### Interstitial telomeric repeats in the *B. antipoda* genome are methylated to a different extent

No unequivocal data about methylation are available for ITRs. The pilot analysis of *A. thaliana* telomeric chromatin (Vrbsky et al., 2010) did not, *a priori*, distinguish between terminal and ITRs. However, high stringency hybridization conditions during their analyses of telomeric cytosine methylation suggested a predominant detection of perfect repeats in genuine telomeres. In another study, methylated cytosines were detected only at ITRs (Vaquero-Sedas et al., 2011). Here, we used two methods to evaluate general and specific patterns of methylation of cytosines located at internal telomeric repeats of *B. antipoda*: (i) hybridization of bisulfite-modified DNA with the DEGENER probe, and (ii) bisulfite sequencing of selected telomeric repeat-containing intrachromosomal regions.

**Figure 4:**
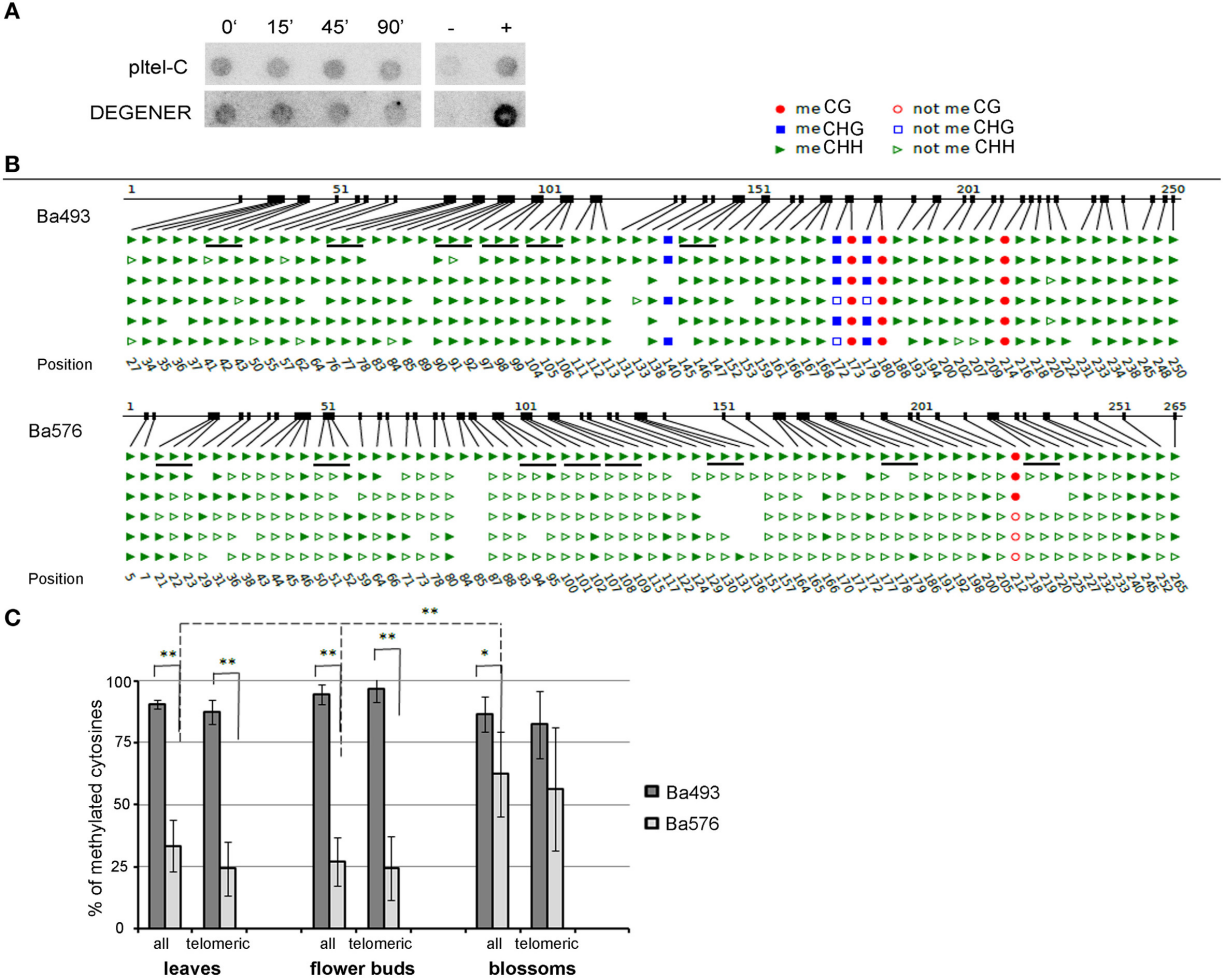
**Methylation of cytosines of *B. antipoda* telomeric repeats. (A)** Analysis of global methylation of cytosines in telomeric repeats of *B. antipoda*. High molecular weight DNA was treated with Bal31, converted with bisulfite and analyzed by Southern hybridization against loading and DEGENER probes. Signals with the DEGENER probe indicating methylated cytosines in telomeric repeats were obtained in all samples. The time of Bal31 digestion is given above the membranes. +, positive hybridization control (tobacco DNA without the bisulfite treatment); −, negative control (DNA from pUC19 plasmid). **(B)** An example of bisulfite sequencing data for Ba493 and Ba576 regions in adult leaves. Non-symmetrically located cytosines are depicted as green triangles; full triangle, methylated; empty triangle, non-methylated cytosine. Cytosines located in perfect telomeric repeats are underlined. The first line of the scheme where all cytosines in all sequence contexts are presented as full figures represents position of respective cytosine. **(C)** Graphical representation of telomeric cytosine methylation within the ITR-containing sequences Ba493 and Ba576. Three tissues were analyzed to reveal possible tissue-specific methylation pattern. “all,” all cytosines located in non-symmetrical sequence context; “telomeric,” cytosines located in perfect telomeric repeats. Data were processed by One-Way independent ANOVA test with standard weighted-means, ^*^ is used for *P* < 0.05, ^**^ for *P* < 0.01.

Dot blot analyses using bisulfite-treated DNA and radioactively labeled DEGENER and loading probes clearly showed that cytosines of *B. antipoda* telomeric repeats were methylated (Figure 4A). The hybridization signal using the DEGENER probe was persistent after extensive Bal31 digestion, implying the presence of methylated cytosines in internally located telomeric repeats that were resistant to the Bal31 treatment. In addition, the intensities of hybridization signals using the pltel-C probe were maintained in Bal31-digested samples, which is in agreement with the data presented in the Figure 2 and confirms localization of a majority of telomeric repeats at internal positions.

We carried out bisulfite sequencing of two ITR regions—Ba493 and Ba576. DNAs from leaves, flower buds, and blossoms were converted by sodium bisulfite and PCR amplified. We found that 87, 96, and 82% of cytosines of telomeric repeats located in the Ba493 region were methylated in leaves, flower buds, and blossoms, respectively (Figures 4B,C), while 24% of cytosines of telomeric sequences of the Ba576 region were methylated in leaves and flower buds and 56% in blossoms (Figures 4B,C). Thus, internal telomeric repeats in *B. antipoda* are methylated and the level of 5mC varies considerably between Ba493 and Ba576 regions which were subjected to the detailed analysis.

### Chromatin of internal and terminal telomeric repeats shows distinct histone marks

Few studies were focused on chromatin features of plant telomeres. In one study, the dual character of *A. thaliana* telomeres with prevalent heterochomatic histone modifications, but clearly detectable euchromatic mark was reported (Vrbsky et al., 2010), and in another, analysis using different techniques demonstrated the predominantly euchromatic character of genuine telomeres and the heterochromatic nature of ITRs (Vaquero-Sedas et al., 2011). We approached this problem from a new perspective and compared histone modifications at terminal and internal telomeric repeats using plants with a different distribution of telomeric repeats between ITRs and genuine telomeres.

**Figure 5:**
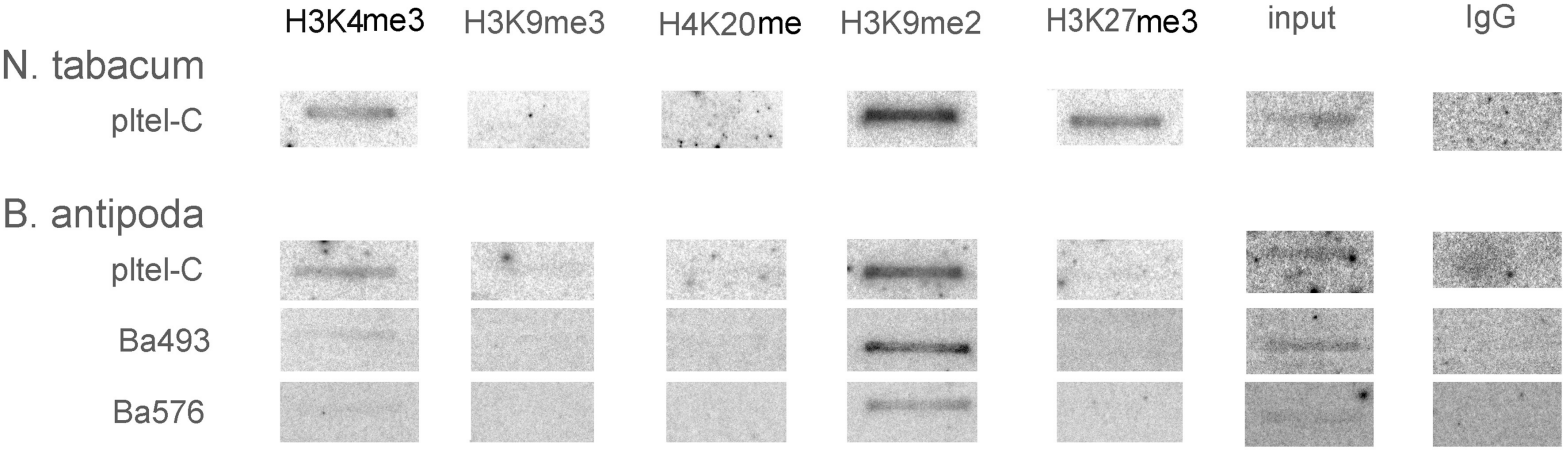
**Analysis of histone modifications at genuine telomeres and ITRs in *N. tabacum* and *B. antipoda***. Chromatin was immunoprecipitated by antibodies against the euchromatic marks H3K4me3 and H3K9me3, heterochromatic marks H4K20me and H3K9me2 and the epigenetic mark for developmentally silenced regions H3K27me3, and hybridized with radioactively labeled probes. In tobacco, H3K9me2 and H3K27me3 signals were prevalent, followed by signal for H3K4me3. *B. antipoda* displayed both heterochromatin- (H3K9me2) and euchromatin-specific (H3K4me3) marks after hybridization with the telomeric pltel-C probe, but only the heterochromatic H3H9me2 mark was clearly above the detection limit after hybridization with the ITR probes Ba493 and Ba576.

To investigate the epigenetic patterns of telomere repeat-containing chromatin at distinct genomic locations, ChIP analyses were carried out using antibodies against five specific histone modifications: H3K4me3 and H3K9me3 as euchromatic marks in plants, H4K20me1 and H3K9me2 as heterochromatic marks, and H3K27me3 as mark of developmentally silenced genes (reviewed in Berr et al., 2011). The telomere-specific probe pltel-C was applied for hybridization to address telomeric sequences; in *B. antipoda* additionally, Ba493 and Ba576 were used to address ITR regions. In accordance with the previous results for *A. thaliana* (Vrbsky et al., 2010), we found that tobacco telomeres were significantly enriched in H3K9me2 and H3K27me3 modifications, with a contribution of euchromatic H3K4me3 (Figure 5). Telomeric repeats of *B. antipoda* showed predominantly the heterochromatic mark H3K9me2, and a minor signal for H3K4me3. No H3K27me3 was detected within telomeric chromatin of *B. antipoda*. Hybridization with the ITR-specific probes Ba493 and Ba576 revealed only signals for H3K9me2 (Figure 5). Extensive mapping of chromatin states in *A. thaliana* showed that H3K9me2 and H3K4me3 marks are, in essence, mutually exclusive (Roudier et al., 2011). In this context, the association of both modifications with plant telomeres may indicate the existence of two distinct fractions of telomeric chromatin; the dominant heterochromatic fraction associated with H3K9me2 and the second one that is more euchromatin-like. We did not find any H3K9me3 signal suggesting different roles for H3K9 and H3K4 trimethylations at plant telomeres, although both these modifications frequently co-localized at euchromatic genes in *A. thaliana* (Roudier et al., 2011). Moreover, we did not detect H4K20me1 at tobacco telomeres, which is astonishing because the dominant plant telomeric mark H3K9me2 was found mainly at heterochromatic transposable elements and other repeats, and overlaps significantly with H4K20me1 in *A. thaliana* (Roudier et al., 2011). The high density of H3K9me2 (Roudier et al., 2011) suggests that these marks may occur independently of each other in distinct chromosome regions, and telomeres may represent such regions.

Our results support the hypothesis that genuine telomeres exhibit a dual epigenetic character, with H3K9me2 as the prevalent mark while ITRs are exclusively heterochromatic. The presence of the H3K27me3 at genuine telomeres in tobacco [and its association with telomeric repeats in *A. thaliana* and rice (Vaquero-Sedas et al., 2012)] represents an interesting feature. Its functional significance remains to be elucidated.

### Telomeres and internal telomeric repeats are both transcribed

In 2007, subtelomere-originated RNA repeats UUAGGG, called TERRA (telomere repeat-containing RNA), were detected in human cells, proving that telomeres are actively transcribed (Azzalin et al., 2007). Since then, TERRA transcripts have been found in birds, budding yeast, fission yeast, *A. thaliana* and tobacco (Vrbsky et al., 2010; Majerova et al., 2011b; Bah and Azzalin, 2012). TERRA is believed to associate directly with telomeres and to regulate the maintenance of telomere length in humans *via* negative control of telomerase activity (Azzalin et al., 2007; Ng et al., 2009). Surprisingly, in yeast cells, a different scenario was recently described; telomeric transcripts are preferentially derived from short telomeres and guide telomerase to the chromosome ends from which they originated (Cusanelli et al., 2013). Transcription of the telomeric G-strand is generally less abundant. In humans, a very faint signal of these transcripts was found, in contrast to fission yeast, where C-strand-rich telomeric RNAs were described and named ARIA and antisense ARRET (αARRET) (Bah et al., 2012). In *A. thaliana*, telomere C-strand (ARRET) transcripts were detected and found to be transcribed mostly from ITRs (Vrbsky et al., 2010). Tobacco and *B. antipoda* allow a comparative analysis of telomere repeat transcripts derived from genuine telomeres vs. ITRs.

**Figure 6:**
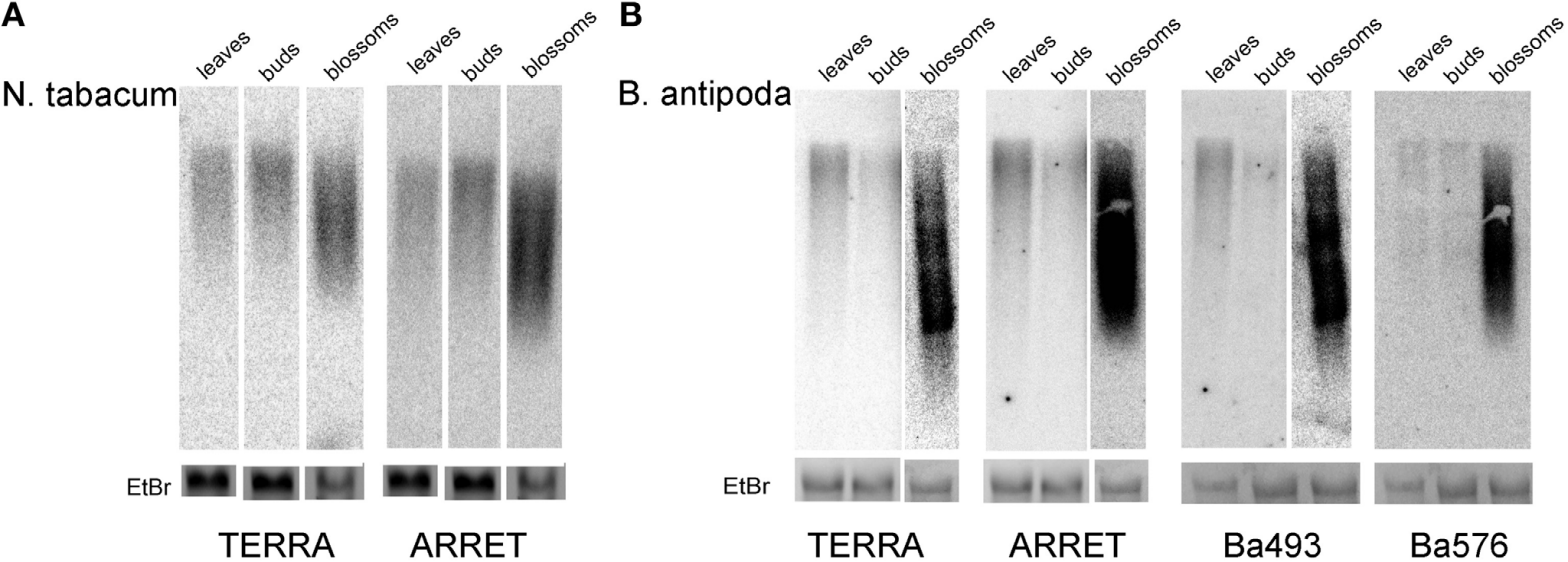
**Northern blot analysis of telomeric RNA in *N. tabacum* (A) and *B. antipoda* (B) tissues**. Transcription of C-rich (TERRA) and G-rich (ARRET) telomeric strands occurred at similar levels in both plants. The lowest levels of TERRA and ARRET were detected in floral buds, followed by leaves and mature flowers, where they were shifted toward shorter fragments. Due to the strong hybridization signal in *B. antipoda* blossom samples, expositions for different time intervals were performed (overnight for leaves and buds, 2 h for blossoms) for TERRA, ARRET and Ba493 probes. EtBr – signal of the 25S ribosomal RNA band at the agarose gel stained by ethidium bromide reflects sample loading. Note, that in none set of samples RNA from blossoms was overloaded.

RNA isolated from leaves of *B. antipoda* and *N. tabacum* plants was analyzed by northern blotting. Membranes were hybridized either with a radioactively labeled pltel-C probe (CCCTAAA)_4_, which hybridizes to the G-rich telomeric strand and detects TERRA, or a pltel-G probe (TTTAGGG)_4_, which hybridizes to the C-rich telomeric strand and detects ARRET. In tobacco, both C-rich and G-rich telomeric transcripts were detected at similar levels (Figure 6A, left lines). Taking into consideration that tobacco has no detectable ITRs (Majerova et al., 2011a), transcription of the G-rich telomeric strand (resulting in ARRET transcripts) can be generated by two possible pathways. According to the first scenario, transcription of the G-rich telomeric strand starts within the telomeres using telomeric repeats or hidden non-telomeric sequences as transcription start sites (TSSs). The second hypothesis assumes synthesis of ARRET directly from TERRA by RNA-dependent RNA polymerases.

To analyze the level of telomeric transcripts in *B. antipoda*, besides pltel-C and pltel-G telomeric probes, specific probes for Ba493 and Ba576 ITR regions were used. No cross-hybridization was detected between Ba493 and Ba576 probes under high stringency conditions (Figure S2A). Therefore, these probes detect the intracellular levels of transcripts derived specifically from the corresponding ITR. Also with tobacco RNA Ba493 and Ba576 probes yielded no signal (Figure S2B). Thus, the probes for *B. antipoda* ITR did not hybridize to telomeres in general.

Both TERRA and ARRET transcripts were detected in *B. antipoda* leaves, at approximately the same level (Figure 6B, left lines). Re-hybridization of membranes with Ba493 and Ba576 probes revealed the presence of transcripts derived from ITR regions. These results imply that a detectable fraction of TERRA and ARRET in *B. antipoda* is transcribed from ITRs. This corresponds with previous results showing that a relevant fraction of telomeric transcripts in *A. thaliana* is derived from ITRs (Vrbsky et al., 2010). We suggest that in plants with a considerable proportion of ITRs in the genome, the telomere repeat-containing RNA originates mostly from the ITRs and only a small fraction of RNA may come from genuine telomeres. Nevertheless, in plants without detectable ITRs, e.g., tobacco, telomeric transcripts must originate from genuine telomeres and, in the case of ARRET, transcription must start within the telomere itself. Whether there is a functional difference between TERRA and ARRET remains to be explored, but a possible role of ARRET may be plant-specific as ARRET was not detected in mammals (Azzalin et al., 2007). In this context, in *A. thaliana*, telomeric transcripts were shown to be involved in RNA-directed methylation of asymmetrically located telomeric cytosines (Vrbsky et al., 2010), and double-stranded RNA molecules participated in this process.

### The level of telomere repeat-containing RNA is tissue-specific and not correlated with methylation of cytosines in ITRs

To quantify transcripts from telomeric repeats in different tissues, RNA samples isolated from leaves, flower buds, and blossoms were analyzed by northern hybridization. Our results showed that the level of telomere repeat-derived RNAs was tissue-specific in both species and no significant differences were found when comparing different transcripts in the same tissue (TERRA vs. ARRET, Ba493 vs. Ba576, Ba576 vs. TERRA, etc., Figure 6). The amount of telomeric RNA in blossoms was an order of magnitude higher than in leaves. Relatively low levels of telomeric transcripts detected in leaves are surprising because similar analyses of *A. thaliana* tissues yielded the highest TERRA level in leaves (Vrbsky et al., 2010). The amount of Ba493 and Ba576 ITR-derived RNA is very similar (Figure 6B) although DNA methylation in these regions differs markedly (see Figures 4B,C). The Ba493 ITR region was shown to be highly methylated (~85% of cytosines are methylated), whereas Ba576 ITR is methylated to about 25% in leaves and floral buds and 56% in blossoms. Differences in levels of telomeric RNA in different tissues are thus not correlated with the level of DNA methylation. The different amounts of RNA could be caused by RNA decay that may vary in different tissues, but no relevant analyses have been done, to our knowledge. Another possibility would be a DNA methylation-independent tissue-specific transcription at the ITR. In tobacco culture cells, the TERRA level was increased in cells with hypomethylated DNA but slightly decreased in cells treated with a general inhibitor of methylation, i.e., in cells with impaired DNA and histone methylation, indicating that the level of TERRA is sensitive to changes in the global methylation pattern (Majerova et al., 2011b). In this context, TSSs located in tobacco genuine telomeres may be more sensitive to DNA methylation [DNA hypomethylation thus increases transcription from tobacco telomeres (Majerova et al., 2011b)], while ITRs in the *B. antipoda* genome are covered with 5mC that do not affect transcript elongation. This hypothesis is testable by further studies, especially in the light of our assumption that tobacco telomeres contain TSSs of unknown sequence(s).

## Conclusion

Localization of telomeric repeats in both terminal and internal chromosomal positions in common model plants significantly complicates interpretation of analyses of epigenetic status of telomeric chromatin, and separate analyses of telomeres and ITRs are methodically not trivial. Our data using model plants with dominant fraction of telomeric repeats at different chromosomal positions revealed that (i) cytosines located in genuine telomeres and ITRs are methylated with the gradient toward distal part of telomeres and different level of methylation in different ITRs, respectively; (ii) telomeres are associated with histones with both euchromatin- and heterochromatin-specific modifications with yet unknown importance for telomere proper structure and function while ITRs are exclusively heterochromatic; and (iii) telomeres and ITRs are transcribed, the level of telomeric transcripts is tissue-dependent, peaks in blossoms and is not correlated with ITRs methylation. These results are beneficial for confirmation and refining of the data obtained using common model plants where the unambiguous interpretation may be influenced by the presence of telomeric repeats at both chromosomal locations. In addition, results demonstrate the usefulness of less common plant models for epigenetic studies.

## Author contributions

Eva Majerová analyzed cytosine methylation, histone modifications, and telomeric transcripts level; Terezie Mandáková performed FISH analyses; Giang T. H. Vu carried out preparation of *B. antipoda* samples for next generation sequencing and participated at the data evaluation; Miloslava Fojtová, Jiří Fajkus, and Martin A. Lysak designed research; Miloslava Fojtová, Jiří Fajkus, Eva Majerová, and Terezie Mandáková evaluated data and wrote the manuscript. All authors read and approved the final version of the manuscript.

### Conflict of interest statement

The authors declare that the research was conducted in the absence of any commercial or financial relationships that could be construed as a potential conflict of interest.
