# Supplementary material for: Chromatin features of plant telomeric sequences at terminal vs. internal positions
Source: Front Plant Sci. 2014 Nov 4;5:593. doi: 10.3389/fpls.2014.00593 (PMC4219495; doi:10.3389/fpls.2014.00593)
Supplement: Supplementary file 1 [file Image1.PDF]

Name	Sequence	Length of the product
pltel-G	5' - TTT AGG GTT TAG GGT TTA GGG TTT AGG G - 3'	
pltel-C	5'-CCC TAA ACC CTA AAC CCT AAA CCC TAA A- 3'	
DEGENER	5' -TTA GRR TTT AGR RTT TAG RRT TTA GRRT- 3'	
493 fwd	5' - AGG ATT ATA AAY YYT AAA AYT TTA AAY TTA AA - 3'	251 bp
493 rev	5' - TTA TCA AAA ATT ATC TCT CCA TTR RTT TTA T - 3'	
576 fwd	5' - AAT TCT CTT AGG ATA ATA AAY YYT AAA - 3'	264 bp
576 rev	5' - AAT RAT CTC TCC ATT TRT TTT ATG ACA AGA TTA A - 3'	

Table S1. List of used primers.

Figure S1

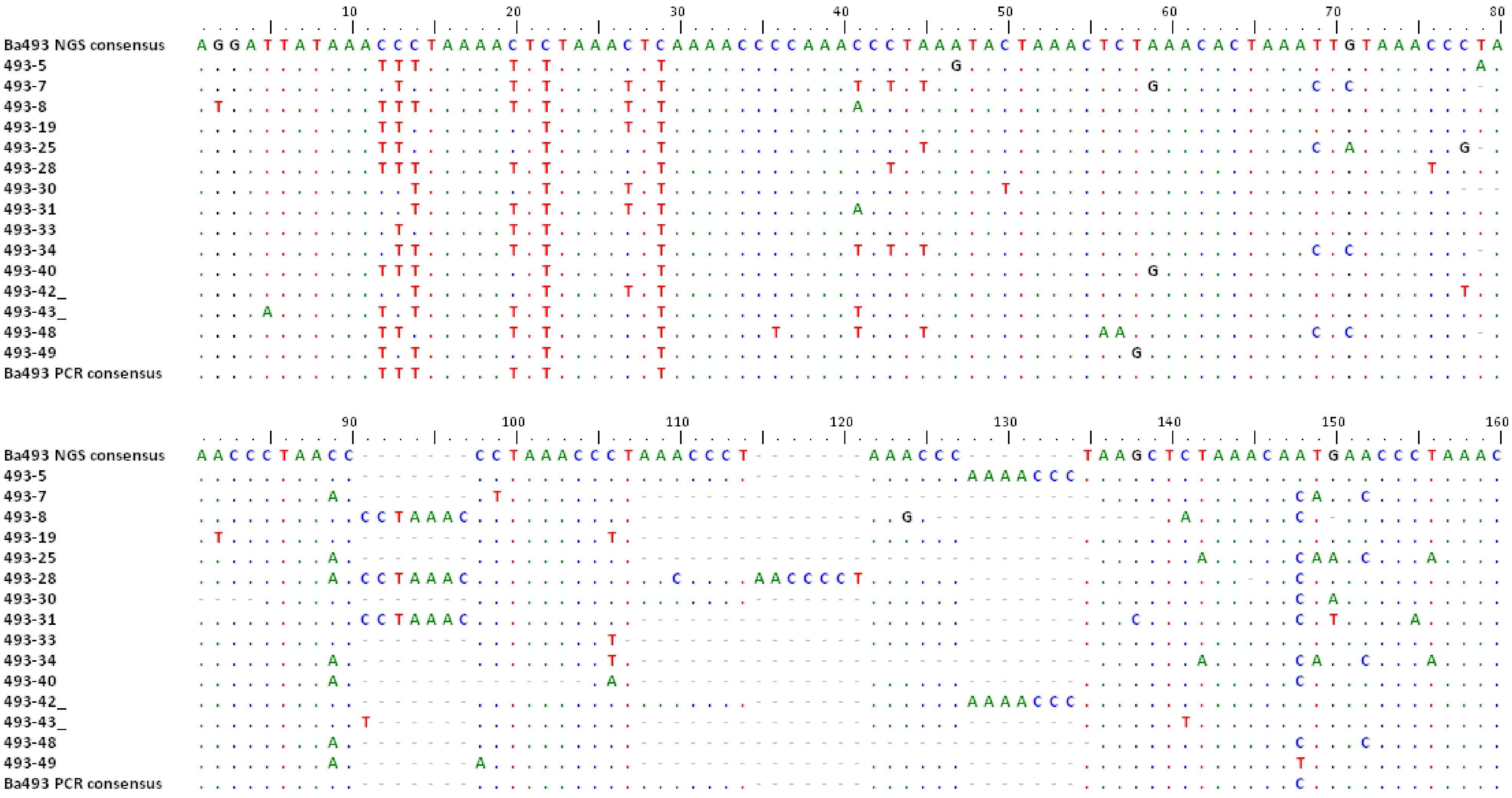
10 20 30 40 50 60 70 80
Ba493 NGS consensus
493-5
493-7
493-8
493-19
493-25
493-28
493-30
493-31
493-33
493-34
493-40
493-42_
493-43_
493-48
493-49
Ba493 PCR consensus
90 100 110 120 130 140 150 160
Ba493 NGS consensus
493-5
493-7
493-8
493-19
493-25
493-28
493-30
493-31
493-33
493-34
493-40
493-42_
493-43_
493-48
493-49
Ba493 PCR consensus

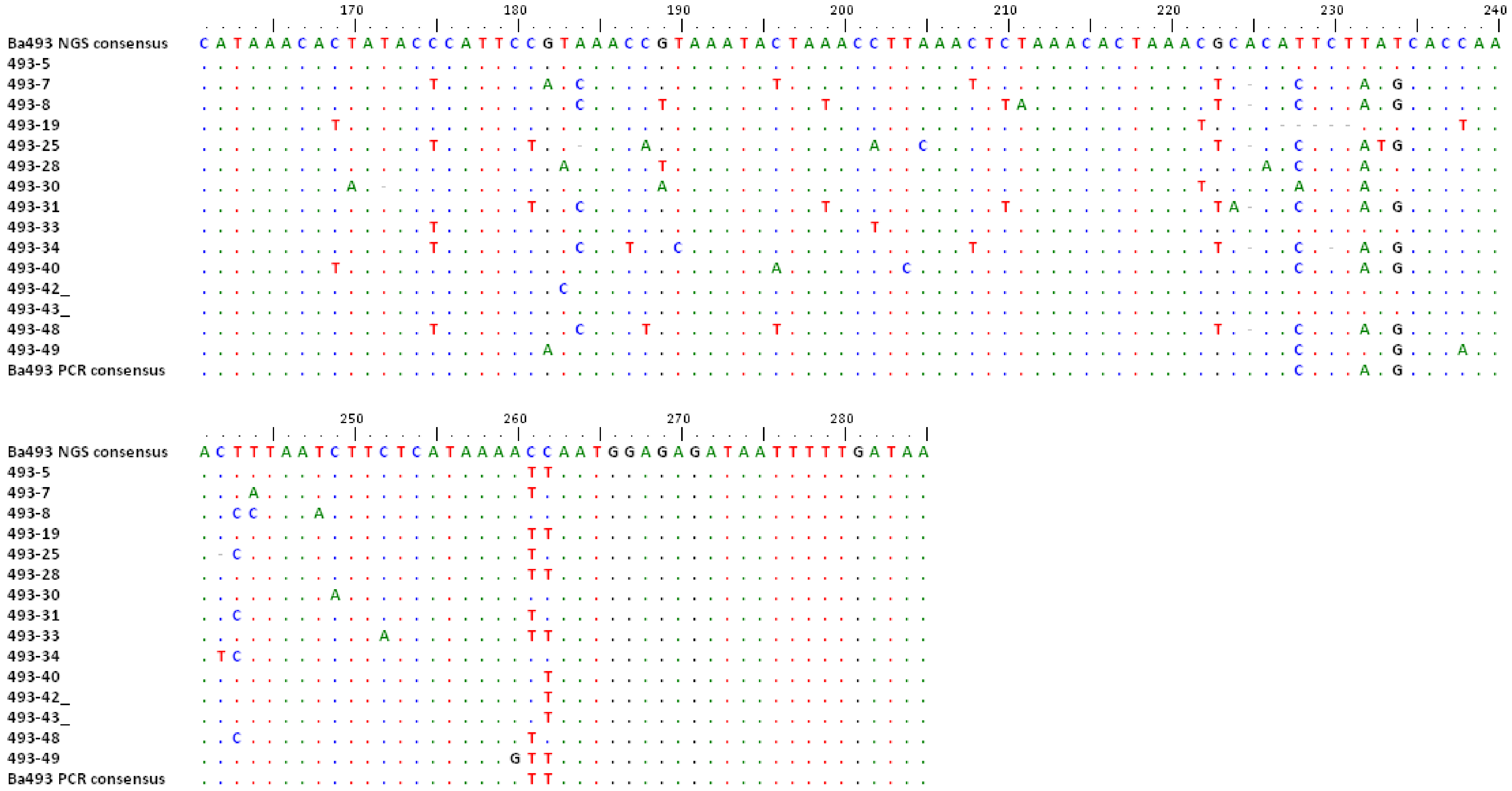
170
180
190
200
210
220
230
240
Ba493 NGS consensus
493-5
493-7
493-8
493-19
493-25
493-28
493-30
493-31
493-33
493-34
493-40
493-42_
493-43_
493-48
493-49
Ba493 PCR consensus
250
260
270
280
Ba493 NGS consensus
493-5
493-7
493-8
493-19
493-25
493-28
493-30
493-31
493-33
493-34
493-40
493-42_
493-43_
493-48
493-49
Ba493 PCR consensus

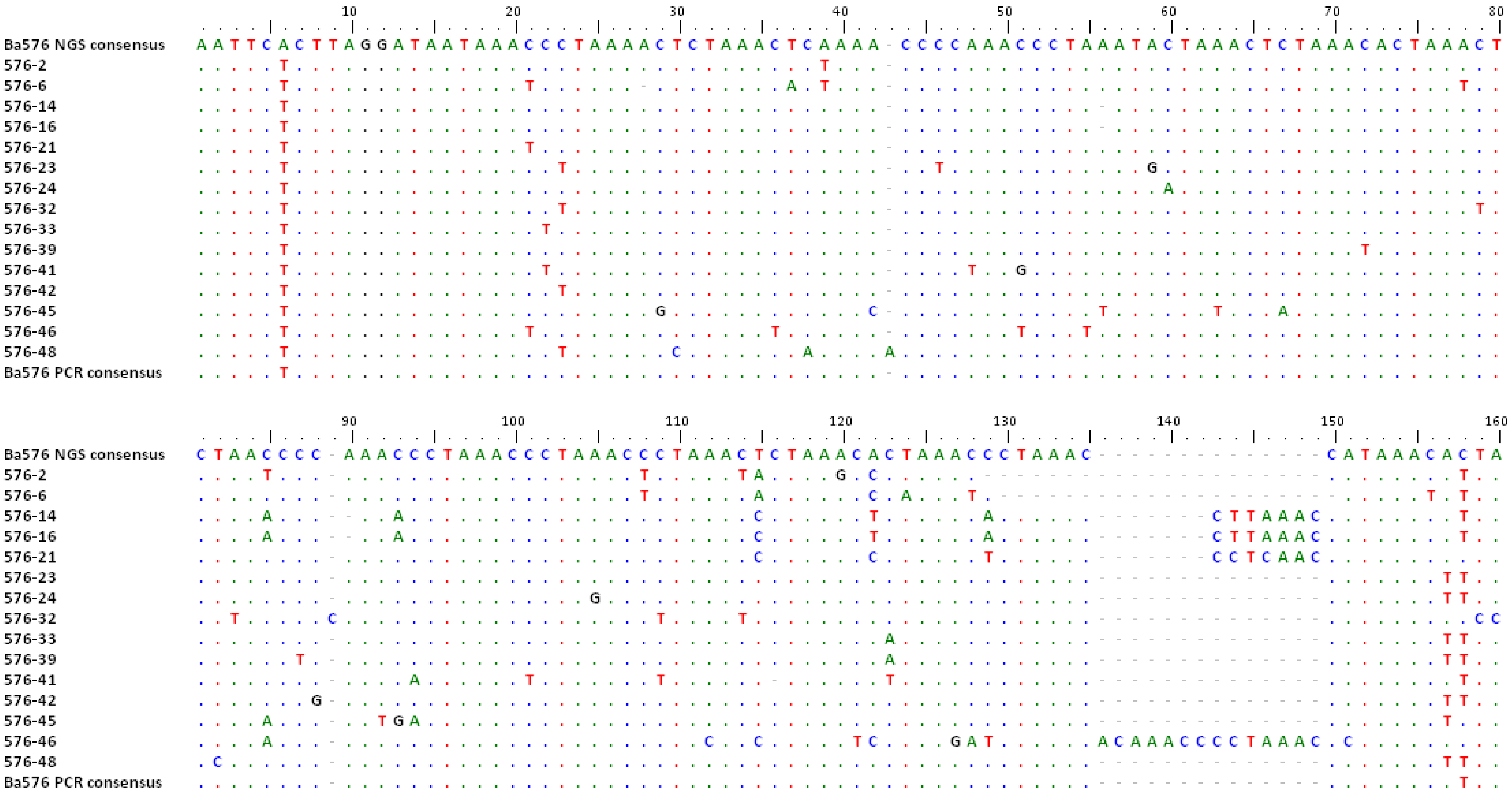
Ba576 NGS consensus
AATTCACTTAGGATAATAAACCCTAAAACTCTAAACTCAAAA-CCCCAAACCCTAAATACTAAACTCTAAACACTAAACT
CTAACCCC-AAACCCTAAACCCTAAACCCTAAACTCTAAACACTAAACCCTAAAC-------------CATAAACACTA
576-2
576-6
576-14
576-16
576-21
576-23
576-24
576-32
576-33
576-39
576-41
576-42
576-45
576-46
576-48
Ba576 PCR consensus

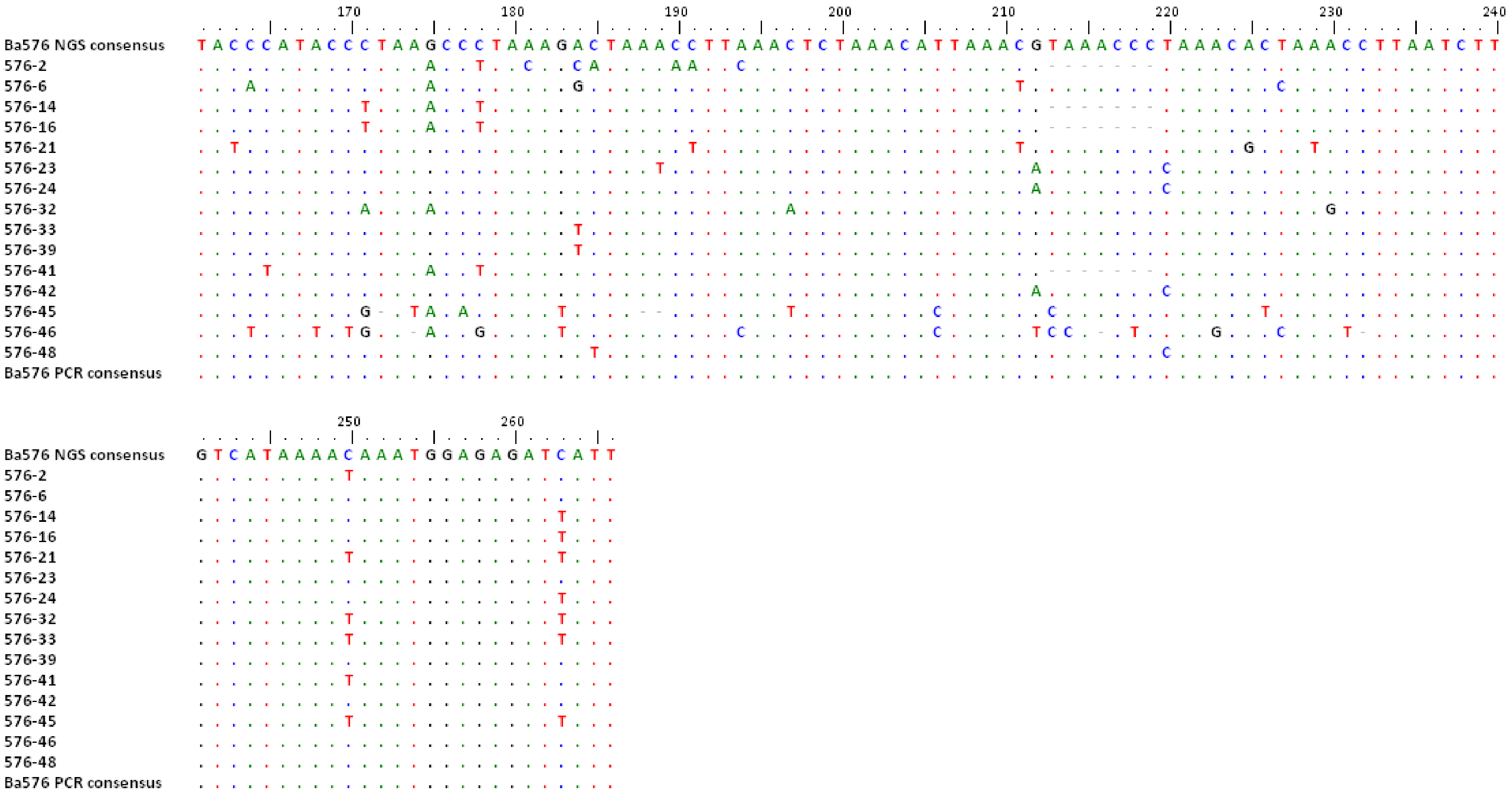
170 180 190 200 210 220 230 240
Ba576 NGS consensus TACCCATACCCTAAGCCCTAAAGACTAAACCTTAAACTCTAAACATTAAACGTAAACCCTAAACACTAAACCTTAATCTT
576-2
576-6
576-14
576-16
576-21
576-23
576-24
576-32
576-33
576-39
576-41
576-42
576-45
576-46
576-48
Ba576 PCR consensus
250 260
Ba576 NGS consensus GTCATAAAACAAATGGAGAGATCATT
576-2
576-6
576-14
576-16
576-21
576-23
576-24
576-32
576-33
576-39
576-41
576-42
576-45
576-46
576-48
Ba576 PCR consensus

Figure S1: Alignment of Ba493 and Ba576 consensus sequences and sequences of clones obtained by amplification of *B. antipoda* genomic DNA. Primers 493 Fw, 493 Rev and 576 Fw, 576 Rev, respectively were used. Frequent C / T transitions were observed. Ba493/576 NGS consensus, consensus sequence from next generation sequencing data; Ba493/576 PCR consensus, consensus sequence based on the sequences of PCR clones.

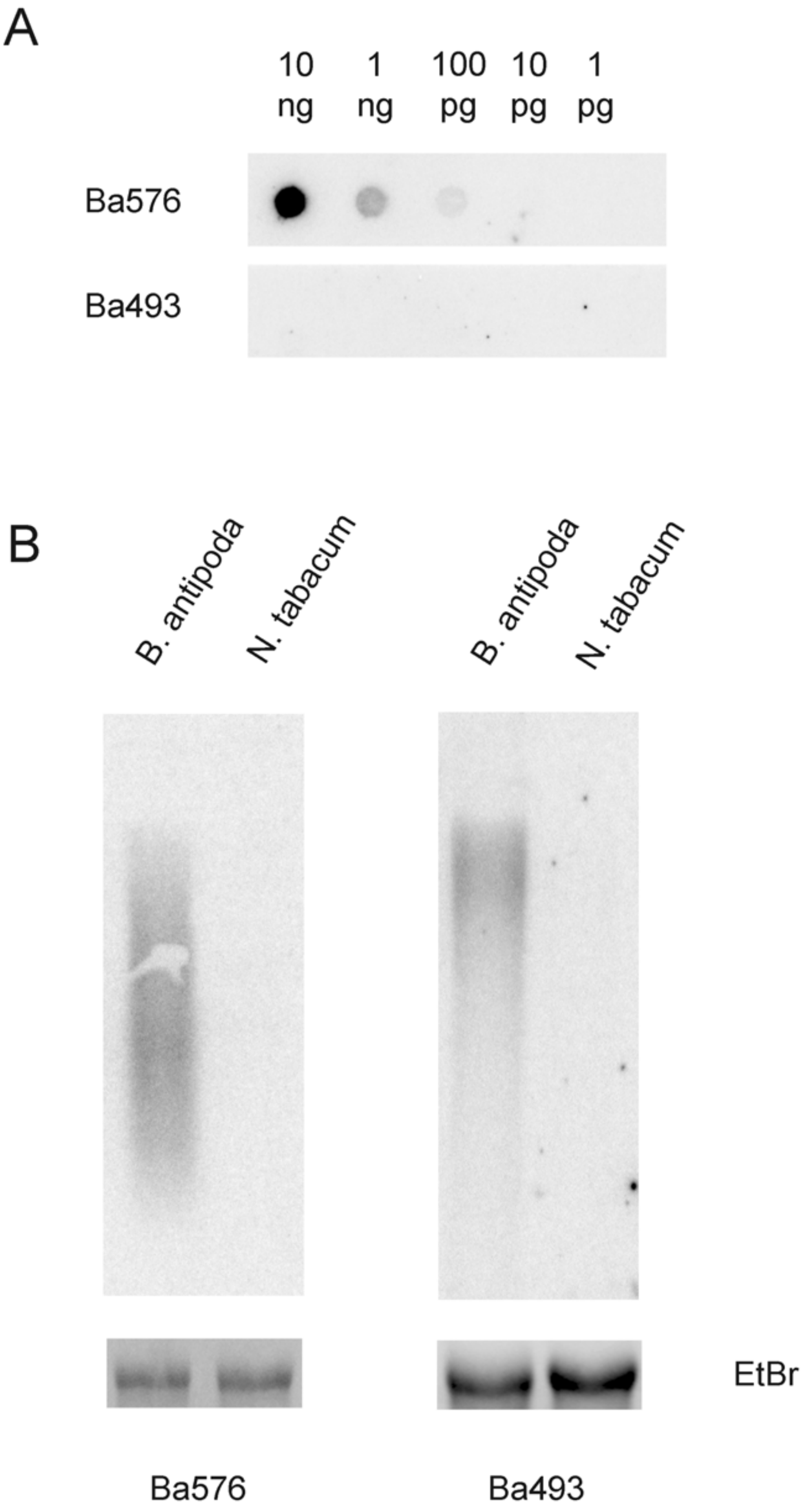

A
10 ng
1 ng
100 pg
10 pg
1 pg
Ba576
Ba493
B
B. antipoda
N. tabacum
B. antipoda
N. tabacum
EtBr
Ba576
Ba493

Figure S2: Specificity of hybridization using Ba493 and Ba576 probes.

(A) Ba493 BAC clone and Ba576 PCR product were dot-blotted onto the nylon membrane (the amount of DNA is given above dots) and hybridized with radioactively labeled Ba576 probe. After washing under high stringency conditions, no signal in Ba493 dots was detected.

(B) RNA isolated from *N. tabacum* and *B. antipoda* leaves was subjected to northern blot and hybridized with radioctively labeled Ba576 (left panel) and Ba493 (right panel) probes. Only in *B. antipoda* lines hybridization signals were detected. EtBr – signal of the 25S ribosomal RNA band at the agarose gel stained by ethidium bromide reflects sample loading.
